# Population pharmacokinetics of intravenous daptomycin in critically ill patients: implications for selection of dosage regimens

**DOI:** 10.3389/fphar.2024.1378872

**Published:** 2024-05-02

**Authors:** Jianhua Wu, Xiangyi Zheng, Liu Zhang, Jiajun Wang, Yifei Lv, Yujie Xi, Dongfang Wu

**Affiliations:** Department of Pharmacy, Zhongnan Hospital of Wuhan University, Wuhan, China

**Keywords:** daptomycin, population pharmacokinetics, nonlinear mixed-effects model, dose selection, critically ill patients

## Abstract

Daptomycin is gaining prominence for the treatment of methicillin-resistant *Staphylococcus aureus* infections. However, the dosage selection for daptomycin in critically ill patients remains uncertain, especially in Chinese patients. This study aimed to establish the population pharmacokinetics of daptomycin in critically ill patients, optimize clinical administration plans, and recommend appropriate dosage for critically ill patients in China. The study included 64 critically ill patients. Blood samples were collected at the designated times. The blood daptomycin concentration was determined using validated liquid chromatography-tandem mass spectrometry. A nonlinear mixed-effects model was applied for the population pharmacokinetic analysis and Monte Carlo simulations of daptomycin. The results showed a two-compartment population pharmacokinetic model of daptomycin in critically ill adult Han Chinese patients. Monte Carlo simulations revealed that a daily dose of 400 mg of daptomycin was insufficient for the majority of critically ill adult patients to achieve the anti-infective target. For critically ill adult patients with normal renal function (creatinine clearance rate >90 mL/min), the probability of achieving the target only reached 90% when the daily dose was increased to 700 mg. For patients undergoing continuous renal replacement therapy (CRRT), 24 h administration of 500 mg met the pharmacodynamic goals and did not exceed the safety threshold in most patients. Therefore, considering its efficacy and safety, intravenous daptomycin doses are best scaled according to creatinine clearance, and an increased dose is recommended for critically ill patients with hyperrenalism. For patients receiving CRRT, medication is recommended at 24 h intervals.

## Introduction

Daptomycin is a cyclic lipopeptide antibiotic with novel antimicrobial targets and rapid bactericidal activity against a wide range of Gram-positive bacteria (Silverman et al., 2003; Hobbs et al., 2008), including methicillin-resistant *Staphylococcus aureus* (MRSA) (Bush et al., 1989) and vancomycin-resistant enterococci (Amsterdam et al., 1994; Metzger et al., 2009; Mohr et al., 2009). It has been approved for the treatment of skin and soft tissue infections and bacteremia, including cases associated with right-sided infective endocarditis (Heidary et al., 2018). The European Society for Clinical Microbiology and Infectious Diseases states in the empirical treatment of MRSA infections that daptomycin should be chosen as the first-line treatment when renal impairment or fatal sepsis is present in patients (Gould et al., 2011). Meanwhile, in the treatment of persistent MRSA bacteremia, daptomycin is more effective if the minimalv inhibitory concentration (MIC) of vancomycin is over 1 μg/mL (Moore et al., 2012; Murray et al., 2013).

Daptomycin is a hydrophilic drug with a small distribution volume (0.1 L/kg) and a high protein binding rate (90%–93%) (Benvenuto et al., 2006; Gregoire et al., 2021). Daptomycin is not metabolized by liver enzymes (Dvorchik et al., 2004) and is mainly eliminated by the kidneys (78%) (Enoch et al., 2007), with a half-life of approximately 8 h (Hawkey, 2008). Daptomycin exhibits concentration-dependent bactericidal activity (Safdar et al., 2004) and its area under the curve (AUC)/MIC ratio is the most relevant pharmacokinetic/pharmacodynamic (PK/PD) index of efficacy (Louie et al., 2001). According to available studies, daptomycin AUC_24h_/MIC ≥666 can be used as a target for therapeutic success against *S. aureus* infections (Falcone et al., 2013), C_min_ can be used to monitor the muscle toxicity of daptomycin with a target value less than 24.3 mg/L (Tally and DeBruin, 2000; Bhavnani et al., 2010), and AUC_24h_/MIC <1422 has also been studied as a safety threshold (Xie et al., 2020).

Critically ill patients are at a high risk of developing life-threatening infection (Dimopoulos et al., 2013) that could result in multiple organ derangements and sepsis (Blot et al., 2014), and are therefore often treated with a combination of organ-supportive therapies, such as continuous renal replacement therapy (CRRT) and extracorporeal membrane oxygenation (ECMO). The PK/PD properties of antibiotics are affected by pathophysiological changes in critically ill patients caused by various factors, such as increased distribution volume (Blot et al., 2014), augmented renal clearance (Udy et al., 2010), altered plasma protein binding (Smith et al., 2012), excretion via CRRT (Kielstein et al., 2010), and sequestration in the ECMO circuit (Shekar et al., 2015). There are no definitive conclusions regarding the dosage of daptomycin administered to critically ill patients; the dosages recommended by various authors range from 6 mg/kg every 48 h to 10 mg/kg every 24 h (Gregoire et al., 2021); some authors have also suggested that different doses of daptomycin should be used at different pathophysiological stages, which may be due to the large PK variability in critically ill patients and the small sample size of previous studies. In this case, a population pharmacokinetic (PPK) model can be used to identify and quantify the variability and then determine the optimal medication administration regimen for each patient. Hence, we collected data from 64 patients at 737 sampling points to develop a PPK model for critically ill Han Chinese patients and seek other appropriate drug recommendations.

## Materials and methods

### Patients and ethics

This study was approved by the Ethics Committee of Zhongnan Hospital of Wuhan University (No. LYL2019018). Informed consent was obtained from the legal representatives of the patients before enrolment.

Plasma samples were collected from patients who received an intravenous infusion of daptomycin in the intensive care unit (ICU) of Zhongnan Hospital of Wuhan University from April 2021 to December 2022 according to the inclusion and exclusion criteria and the clinical sample sampling protocol for blood drug concentration testing. Patients who fulfilled the following criteria were eligible: ≥18 years old; treated with daptomycin for proven/probable infections caused by Gram-positive organisms that were unresponsive or intolerant to other antimicrobial agents. The exclusion criteria were pregnancy, known or suspected hypersensitivity to daptomycin and its excipients, and incomplete or missing laboratory data before and after treatment.

Demographic and clinical characteristics of the enrolled patients were also collected from the medical records, including age, sex, weight, diagnosis, comorbidities, outcome, combination of other drugs (including antimicrobials), daily complete blood counts, biochemical test results (including liver and renal function and creatinine phosphokinase (CPK)), and whether CRRT or ECMO therapy was performed. In addition, acute physiology and chronic health evaluation (APACHE) and sequential organ failure assessment (SOFA) scores were calculated for each patient to determine disease severity. The patient’s creatinine clearance (CL_CR_) was calculated according to the Cockcroft-Gault formula based on serum creatinine at steady state on day 3.

### Daptomycin administration and sample collection

Daptomycin (Jiangsu Hengrui Medicine Co., Ltd.) dissolved in 100 mL normal saline was administered as a 30-min infusion. The daptomycin dose was 500 mg every 24 h. To obtain a complete blood drug concentration time curve after administration, we took into account the equilibrium phase (near peak concentration) and elimination phase of the drug in the design of sampling time points. On the first day of administration, blood samples were collected at 0, 0.5, 1, 2, 4, 8, 12, and 24 h after infusion. On the third day of administration, blood samples were collected before administration and at 0, 0.5, 1, 2, 4, 8, 12, and 24 h after infusion. On the fifth and seventh days, blood samples were collected before administration and at 0.5 and 4 h after infusion. Blood was drawn and allowed to clot in yellow blood-collecting tubes containing separating glue and a coagulant accelerator. Serum was collected by centrifugation and stored at −80°C until assayed. As soon as the study dose was administered, the participants were monitored for potential side effects. Serious adverse events were defined as adverse events related to daptomycin that required treatment discontinuation.

### Measurement of daptomycin serum concentrations

Serum daptomycin concentrations were measured using a high-performance liquid chromatography-tandem mass spectrometry method developed in our laboratory, the lower limit of quantification was 0.05 μg mL^−1^. Briefly, serum samples were analyzed after protein precipitation. Daptomycin-d5 (Beijing Hager Biotechnology Co., Ltd.) was used as an internal standard. A Waters ACQUITY UPLC C8 column (2.1 mm × 50 mm, 1.7 μm) was used for separation. The mobile phase consisted of phase A: water (0.1% formic acid), phase B: 1:1 methanol: acetonitrile (0.1% formic acid) at a flow rate of 0.4 mL min^−1^, column temperature of 45°C, injection volume of 2 μL, and analysis time of 4 min. Ion transitions were performed using positive electrospray ionization in the multiple reaction monitoring mode.

Before measurement, the sample was restored to room temperature, 100 μL of 1:1 methanol: acetonitrile was measured into a 1.5 μL EP tube, then 10 μL of daptomycin-d5 with 40 μL of sample/standard/quality control was added, the sample was vortex shaken for 1 min, centrifuged at high speed for 8 min (14,500 r/min), and the supernatant was placed in a 96-well plate for the assay.

### Daptomycin pharmacokinetic and data analysis

NONMEM (version 8.1; ICON Development Solutions, Hanover, MD, United States) was used to establish a population PK model for daptomycin and to perform Monte Carlo simulations (Bauer et al., 2007). The ADAPT-TRAN program was used for pre- and post-processing (Savic et al., 2009). This study explored one- and two-compartment models of population PK of daptomycin. The two-compartment, disposition model was parameterised in terms of total clearance (CL), volume of distribution in the central compartment (V_C_), volume of distribution in the peripheral compartment (V_P_), and intercompartmental clearance (Q). The first order conditional estimation with inter- and intra-subject variability interaction (FOCE-I) was employed for all model runs. The inter-individual variability (η) was described using lognormal distributions, and a mixed additive and proportional model was chosen for residual variability (Ɛ). Additionally, the importance of the off-diagonal elements of the omega variance-covariance matrix was investigated. We explored the potential effects of candidate covariates on daptomycin disposal. The main variables included body size (body Mass Index [BMI] and body weight [WT]), age, sex, creatinine clearance (CCR), albumin concentration, SOFA and APACHE II scores for daptomycin clearance (CL), and the effects of WT and sex on volume of distribution.

Stepwise screening of the covariates was performed using Perl Speaks NONMEM (version 5.3.0; Uppsala University Pharmacometrics Research Group, SE), including both forward inclusion and backward elimination processes. The level of significance for keeping the covariates in the model was set at 0.05 and 0.01, respectively.

### Model evaluation

The model development and evaluation were guided on the basis of goodness-of-fit plots and parameter estimate precision. Moreover, a visual predictive check (VPC) was carried out to confirm that model simulations could reproduce the observed data. Bootstrap analysis with 1000 samples was used to evaluate the accuracy of the parameter estimation.

### Monte Carlo simulations of dosage regimens

NONMEM software was used to simulate 1000 virtual subjects to assess the inter-subject variability (BSV) with different clinical doses (400, 500, 600, and 700 mg) for half an hour in patients with different renal function levels (creatinine clearance rates were 20, 30, 40, 60, 90, 120 mL/min).

### Statistical analysis

The Kolmogorov-Smirnov test was employed to assess whether data were normally or non-normally distributed. Continuous variables are presented as means ± standard deviations if normally distributed and as median with IQR if abnormally distributed. Categorical variables were compared by the *χ*2 test while continuous variables were compared using the Student’s t-test or Mann-Whitney test.

## Results

### General information and statistics

**FIGURE 1:**
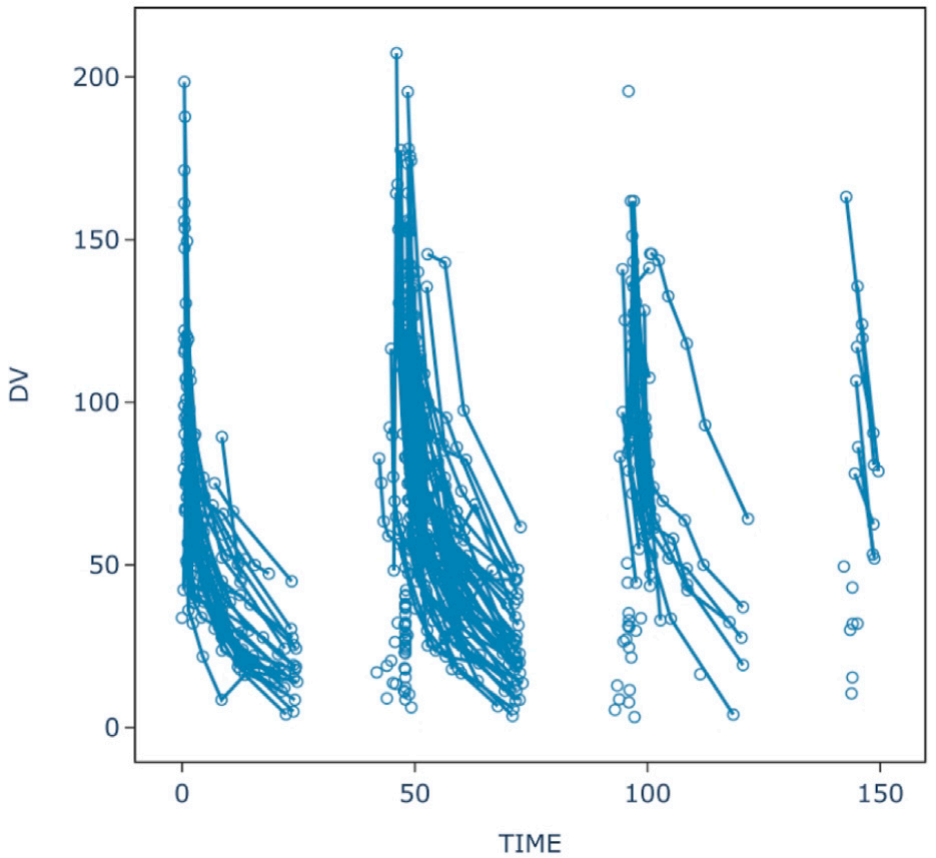
Serum concentration-time profiles of daptomycin in 64 patients.

This study included 64 critically ill patients (43 males and 21 females), with no significant differences found between males and females. The specific information of all patients is shown in Table 1. Sixty-three patients weighed 45–90 kg, whereas the remaining patient was extremely obese (170 kg). Among them, 39 patients received CRRT, and different patients used different hemodialysis filters. Among these, 17 patients used the MultiFiltrate Kit Ci-Ca AV1000S (Fresenius Pharmaceuticals, Bad Homburg vor der Höhe, Germany; membrane area 1.8 m^2^); two patients were treated with Ultraflux AV600S (Fresenius Pharmaceuticals; membrane area 1.4 m^2^), and 20 were treated with Prismaflex M100AN69 (BAXTER Baite International Limited, Jinbao; membrane area 0.9 m^2^). The blood flow rate was maintained between 95 and 350 mL/min, and the effluent flow was maintained between 1600 and 2000 mL/h and replaced as clinically indicated. Among these 45 CRRT patients, four used ECMO in combination. In addition, two patients received ECMO alone. No increase in CPK or other adverse reactions related to daptomycin were observed in any of the patients during the treatment period. A total of 737 blood concentration values were included in the population PK analysis, and the serum concentration-time curves of all blood samples are shown in Figure 1.

**TABLE 1:** Patient characteristics.

Characteristics	n (%) or median (IQR)
Age (years)	57.5 ± 16.5
Sex	
Male	43(67.2%)
Female	21(32.8%)
Weight (kg)	64.5(45–170)
Body mass index (kg/m2)	23.0(16.5–52.5)
Serum creatinine (μmol/L)	106(21.7–845.4)
Creatinine clearance (mL/min)	54.25(8.3–200.2)
With renal replacement therapy	
CRRT	39(60.9%)
No CRRT	25(39.1%)
With ECMO	6(9.4%)
only ECMO	2(3.1%)
ECMO with CRRT	4(6.3%)
Albumin (g/L)	32.8(19.6–46.8)
Total proteins (g/L)	54.2(37.1–573)
Alanine transaminase (U/L)	63(8–2581)
Aspartate transaminase (U/L)	71(11–3845)
Total bilirubin (μmol/L)	35.3(5.2–312)
APACHE II score	24(12–47)
SOFA score	12(4–19)

Categorical variables are denoted as n (%) and the remaining variables are denoted as median (range).

### Pharmacokinetic models and factors affecting them

One- and two-compartment models were fitted separately, and the results of the objective function value (OFV) and goodness-of-fit plots showed that the time course of daptomycin concentration was well described by a two-compartment linear model with linear elimination. Unexplained residual variability was best described by additive and proportional residual error models. CCR had a significant effect on renal clearance in non-CRRT patients. After the inclusion of CCR, no other covariates significantly improved model fit. Sex was associated with individual-specific V_P_, and during the three rounds of forward inclusion, all three ΔOFV values were greater than 3.84; the V_P_ of males was approximately 1.4 times that of females, but in the backward elimination process, the influence of sex was not retained in the final model. The SOFA score was marginally associated with individual-specific CL and was excluded from the backward elimination process. Furthermore, no relationships were identified between the CL, V_C_, or V_P_ of daptomycin and APACHE II score, age, or serum albumin concentration.

The final covariate model is as follows:

$$\text{CL}\left(L/h\right)=\left\{\begin{array}{l} 0.386\text{in subjects undergoing CRRT}\\ 0.229+0.148\cdot \left(\frac{CCR}{54}\right)\text{in}\text{others} \end{array}\right.$$

$$V_{C}\left(L\right)=4.14$$

$$V_{P}\left(L\right)=3.52$$

$$Q\left(L/h\right)=2.09$$

where CCR/54 is the corresponding median standardized individual CCR calculated for the current patient population.

### Model validation and evaluation

**FIGURE 2:**
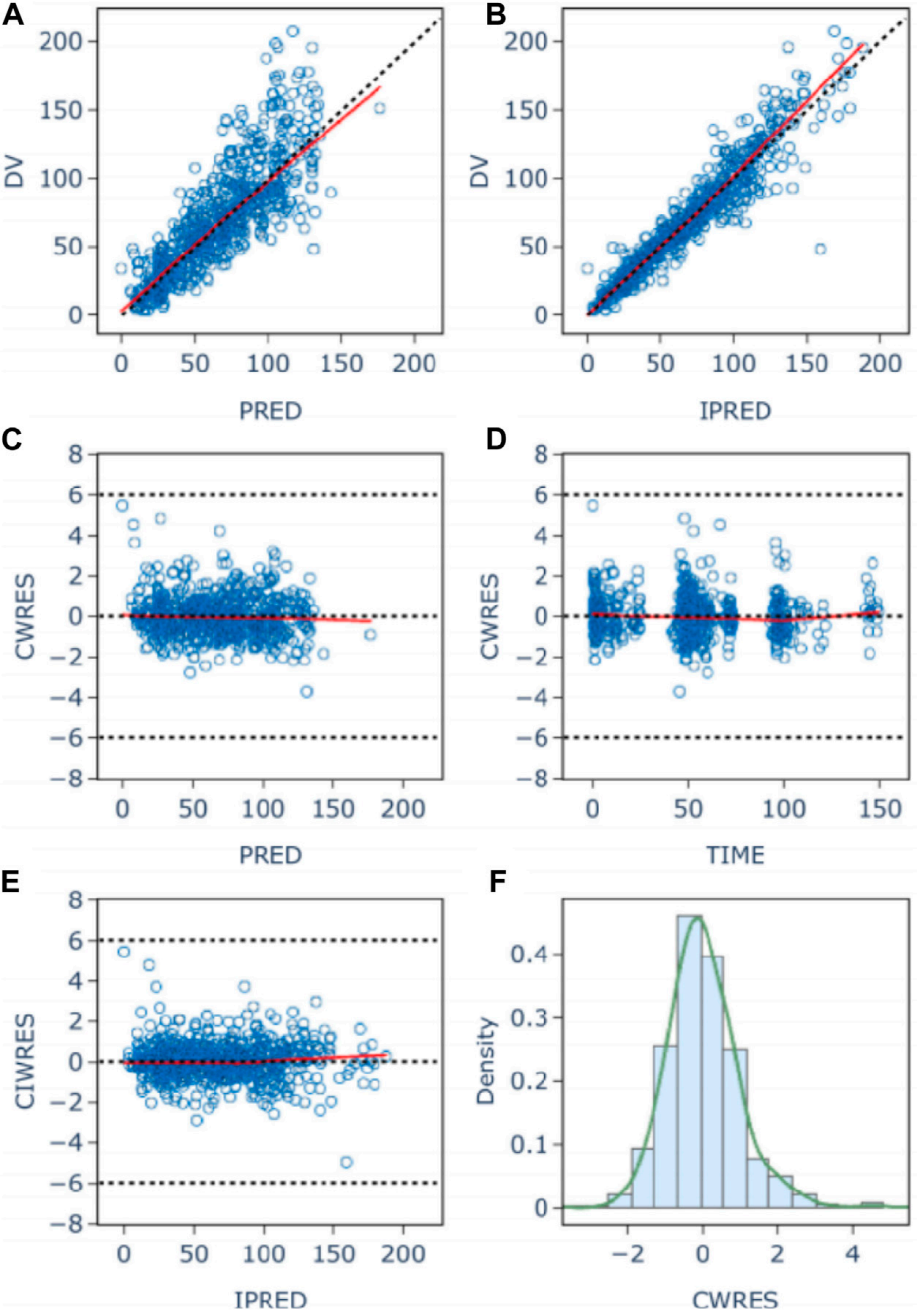
Population **(A)** and individual **(B)** fitted *versus* observed daptomycin concentrations; Diagnostic plot of Conditional weighted residuals (CWRES) *versus* population predicted value **(C)**, time **(D)**, and individual Predicted Values **(E)**; histogram of conditional weighted residuals **(F)**. The red lines display the trend of the data, the dotted black lines indicate the expected trends, the blue circles represent the observed data.

**FIGURE 3:**
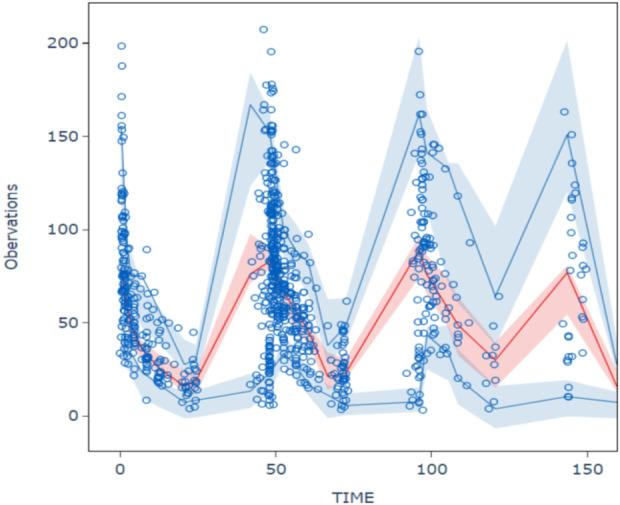
Visual predictive chenk of final model The hollow circles are the actual observations, the red line is the median observation, the upper and lower blue lines correspond to 5% and 95% of the measured values, and the shaded area corresponds to the 90% prediction intervals for the median of the 1,000 simulations *versus* 5% and 95%, respectively.

The goodness-of-fit plots for model checking are presented in Figure 2; the results show that the trend deviation of the final model is unbiased, and the fit is satisfactory. Table 2 lists the parameters of the final population PK models together with the bootstrap results. All PK parameters were statistically significant as the 95% confidence intervals from the bootstrap analysis did not include 0. The VPC graph is shown in Figure 3, The quantile lines of the observed concentrations were all within the 90% confidence interval of the corresponding quantile lines of the predicted concentrations, which indicates a good correlation between raw data and data obtained by simulation with the final model, so the model has good prediction ability.

**TABLE 2:** Final population pharmacokinetic models estimates and bootstrap results for daptomycin.

Parameter	Final Model			Bootstrap		
	Estimate	RSE%	Shrinkage%	Medians	95% Bootstrap CI	
**Theta**						
NR (L/h)	0.229	25.1		0.231	0.125–0.36	
V_C_ (L)	4.14	4.8		4.14	3.763–4.565	
V_P_ (L)	3.52	8.2		3.527	2.987–4.078	
Q (L/h)	2.09	8.2		2.086	1.736–2.482	
CRRT (L/h)	0.386	5.1		0.386	0.351–0.428	
R	0.152	31.3		0.15	0.062–0.264	
**Omega**						
η[CL]	0.091	9.1	6.8	0.085	0.059–0.122	
η[V_C_]	0.114	9.6	11.4	0.11	0.074–0.157	
η[V_P_]	0.202	14.8	29.6	0.193	0.088–0.35	
**Sigma**						
Ɛ[PROP]	0.018	9.3	11.9	0.018	0.012–0.027	
Ɛ[ADD]	38.095	16.1	9.7	38.009	12.368–61.724	
**OFV**	4574.582					
**AIC**	4596.582					
**BIC**	4647.211					

(Percentage of relative error, RSE %) = (standard error, SE)/(final value of parameter estimate, FPE) ×100, NR, no renal clearance; R, renal clearance; η, inter-individual variability; Ɛ, residual variability.

### Monte Carlo simulation

**FIGURE 4:**
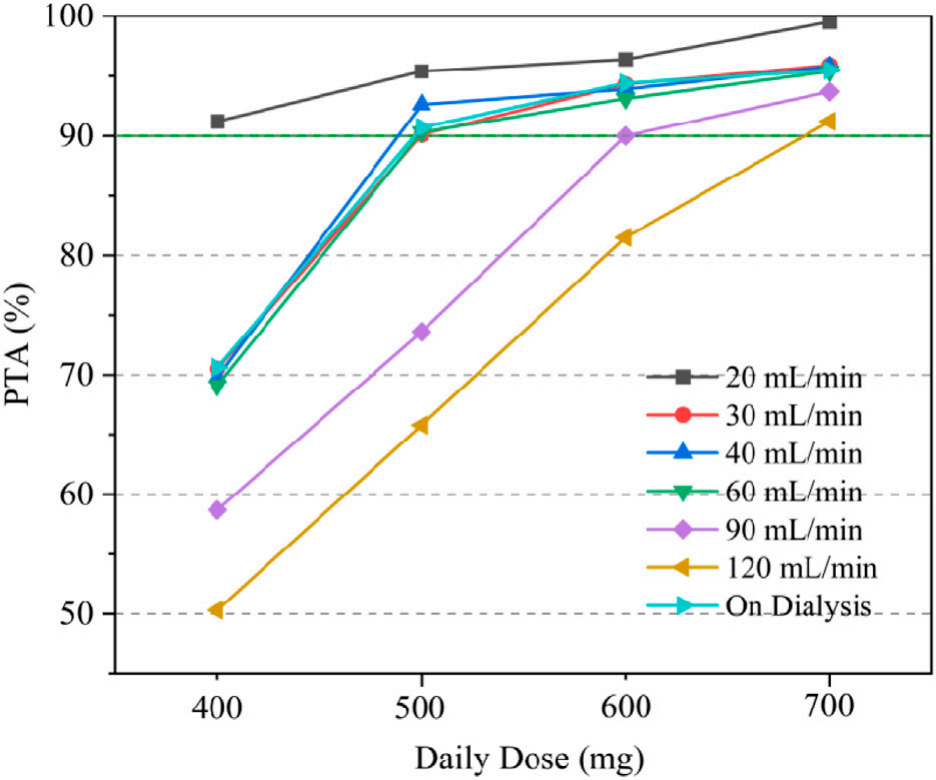
PTA with different dose regimens under different renal function levels.

Due to the lack of clear MIC values for some of the included patients undergoing preventive anti-infection treatment, we took MIC = 1 mg/L for subsequent calculations based on the clinical breakpoints identified by EUCAST for *Staphylococcus* spp. and *Streptococcus* spp. (Gregoire et al., 2021), and probability of reaching target (PTA) was defined as the probability of AUC_24h_/MIC ≥666. Simulations of the dosage regimens in patients with different renal functions are shown in Table 3, and the PTA is shown in Figure 4. When the MIC value was 1 mg/L, the majority of critically ill adult patients were unable to achieve the anti-infective target when the daily dose of daptomycin was 400 mg (equivalent to a dose of 6 mg/kg/day for a patient with a body weight of 70 kg); when the daily dose was 500 mg, the probability of achieving the pharmacodynamic target value reached 90% in adult patients receiving CRRT and with different degrees of renal impairment, and the infection could be better controlled; for critically ill adult patients with normal renal function (CCR ≥90 mL/min), the daily dose needed to be increased to 700 mg (equivalent to the dose of 10 mg/kg/day for patients weighing 70 kg) in order to achieve 90% probability of reaching the pharmacodynamic target value. In patients receiving CRRT, the PTA was only 70.6% when administered at a dose of 400 mg for 24 h. It was necessary to increase the dosage to 500 mg to increase the PTA to 90.7%. Therefore, in patients with normal renal function, especially in critically ill patients with hyperrenalism, it is recommended to increase the dosage from the standard dosage recommended in the daptomycin insert and 500 mg q24 h is recommended for patients receiving CRRT.

**TABLE 3:** Median AUC_24h_ and probability of attainment for each dosing regimen at different levels of renal function.

								
Parameters	Daily Dose (mg)	Value for subjects in CCR group						
		20 mL/min	30 mL/min	40 mL/min	60 mL/min	90 mL/min	120 mL/min	On CRRT
Median AUC_24h_ (μg·h/mL)	400	1145.9	884.2	981.1	817.5	745.4	659.7	884.2
	500	1432.3	1105.3	1226.4	1089.4	943.0	824.6	1105.3
	600	1718.8	1326.3	1471.6	1307.3	1131.6	989.5	1326.3
	700	2005.3	1547.4	1716.9	1525.2	1320.2	1154.4	1547.4
PTA (%)	400	91.2%	70.5%	69.8%	69.1%	58.7%	50.3%	70.6%
	500	95.4%	90.1%	92.6%	90.3%	73.6%	65.8%	90.7%
	600	96.4%	94.3%	93.9%	93.1%	90.0%	81.5%	94.4%
	700	99.5%	95.9%	95.8%	95.4%	93.7%	91.2%	95.5%

Abbreviations: AUC_24h_, area under the plasma concentration-time curve over 24 h; CCR, creatinine clearance.

## Discussion

There were significant individual differences among the critically ill patients. Differences in age, weight, liver and kidney function, disease conditions, treatment methods, and physiological and biochemical indicators may lead to changes in the PK of daptomycin. Therefore, therapeutic drug monitoring (TDM) and precise drug delivery plans based on PPK models are important tools for optimizing the use of antibacterial drugs (Abdulla et al., 2021; Wicha et al., 2021; Oda et al., 2023).

To our knowledge, this is the largest population pharmacokinetic study of daptomycin in critically ill Han Chinese patients. In previous reports on the PPK of daptomycin, some were densely sampled, whereas others were sparsely sampled or directly modeled using blood drug concentration values from TDM. The final models are mostly one- or two-compartment models (Di Paolo et al., 2013; Soraluce et al., 2018; García-Martínez et al., 2022). Our study found that the population pharmacokinetic model of daptomycin in critically ill adult Chinese Han patients can be described by a two-compartment model, in which clearance in non-CRRT patients can be divided into renal and non-renal clearance components, and the renal clearance component is affected by creatinine clearance. By examining the effects of covariates, such as age, weight, sex, and liver and kidney function, on the pharmacokinetics of daptomycin, we found that only CCR significantly affected the renal clearance of daptomycin, consistent with Soralue’s conclusion (Soraluce et al., 2018). The effect of CCR on daptomycin clearance has been extensively documented (Di Paolo et al., 2013; Chaves et al., 2014). Some previous studies found that body weight was associated with clearance. The differing results in this study may be due to the use of the Cockroft-Gault equation to calculate the CCR in the present study, and that the effects of body weight, age, and sex were already included in the CCR; therefore, the effect of body weight on CL was not significant after inclusion of the CCR. The effect of body weight on daptomycin distribution volume was also not observed, and some PPK studies support this conclusion (Di Paolo et al., 2013; Falcone et al., 2013; Soraluce et al., 2018). In addition, we found that V2 was slightly higher in males than in females. Other covariates did not significantly improve the model, and no effect of the combined use of extracorporeal membrane oxygenation on the pharmacokinetics in critically ill adult patients was observed. The final pharmacokinetic parameters are expressed as population values ±standard error as follows: the non-renal clearance rate (CL_NR_) of non-CRRT patients is (0.23 ± 0.05) L/h, and the renal clearance part (CL_R_) is (0.14 ± 0.035) × (CCR/54). The CL_CRRT_ value of patients receiving CRRT is (0.388 ± 0.050) L/h, which is similar to the results reported in previous PK studies of CRRT patients (Khadzhynov et al., 2011; Vilay et al., 2011). V1 is (4.20 ± 0.19) L and V2 is (3.67 ± 0.25) L were higher than in healthy volunteers (Gregoire et al., 2021) and similar to those reported in a previous study on PPK combining healthy participants and patients with different renal functions. The Q is (2.13 ± 0.17) L/h, which was slightly lower than in previous studies (Dvorchik et al., 2004; Soraluce et al., 2018; Xie et al., 2020). This result may be due to differences between the study population and a single sample source.

When optimizing the administration plan of daptomycin, we found that a higher dose is desirable to achieve a high treatment success rate (Safdar et al., 2004; Falcone et al., 2013; Ando et al., 2020) in patients with normal renal function, especially critically ill patients with hyperrenalism. For patients receiving CRRT, the instructions recommend 6 mg/kg q48 h dosing. However, with q48 h dosing, the patient’s exposure to the drug on the second day is lower, and there may be a risk of treatment failure on the second day, especially in patients with sepsis in the ICU (Kielstein et al., 2010; Chaves et al., 2014). Our results showed that for patients receiving CRRT, 500 mg q24 h administration met the pharmacodynamic target and did not exceed the safety threshold in most patients and that despite the presence of a small number of patients with Cmin >24.3 mg/L, no elevation of CPK associated with daptomycin was found (Ando et al., 2020). Several studies have indicated that Cmin >24.3 mg/L does not correlate well with skeletal muscle toxicity. Therefore, considering both efficacy and safety, we recommend q24 h dosing in patients receiving CRRT.

There are some limitations to this study, mainly that all patients were from a single center; therefore, it is hoped that a multicenter study can be conducted to include a larger sample and further optimize the model to better individualize medication.

## Conclusion

Overall, this study provides a PPK model for daptomycin in critically ill Han Chinese patients. Our study showed that intravenous daptomycin doses are best scaled by creatinine clearance; a higher dose is recommended for critically ill patients with hyperrenalism, and 24 h medication is recommended for patients receiving CRRT.

## Data Availability

The original contributions presented in the study are included in the article/Supplementary Material, further inquiries can be directed to the corresponding author.
