# Supplementary material for: Population pharmacokinetics of intravenous daptomycin in critically ill patients: implications for selection of dosage regimens
Source: Front Pharmacol. 2024 May 2;15:1378872. doi: 10.3389/fphar.2024.1378872 (PMC11096781; doi:10.3389/fphar.2024.1378872)
Supplement: Supplementary file 1 [file Table1.docx]

Table S1.Model building process

| Model No. | Description | OFV | ΔOFV | p value |
| --- | --- | --- | --- | --- |
| 1 | base | 5304.788 | - | - |
| 2 | base+CCR on CL | 5286.376 | -18.412 | <0.05 |
| 3 | base+SOFA on CL | 5303.564 | -1.224 | >0.05 |
| 4 | base+GEND on VP | 5299.690 | -5.098 | <0.05 |
| 5 | base+AGE on CL | 5303.157 | -1.631 | >0.05 |
| 6 | base+CCR on CL+GEND on VP | 5281.123 | -5.253 | <0.05 |
| 7 | model6-CCR | 5299.69 | 18.567 | <0.001 |
| 8 | model6-GEND | 5286.376 | 5.253 | >0.001 |
